# A Challenging Case of Metastatic Non-Small Cell Carcinoma of the Lung

**DOI:** 10.7759/cureus.38319

**Published:** 2023-04-30

**Authors:** Annalee Mora, Amirali Ghavamrevaii, Omar Antabli, Ali Vaziri

**Affiliations:** 1 Internal Medicine, Hospital Corporation of America (HCA) Healthcare/University of South Florida (USF) Morsani College of Medicine Graduate Medical Education (GME) Oak Hill Hospital, Brooksville, USA

**Keywords:** quality-of-life, adenocarcinoma, non-small cell carcinoma, metastasis, : lung cancer

## Abstract

Primary lung carcinoma with distant metastasis is a life-threatening diagnosis that presents many unique challenges due to the severity of the disease at the time of presentation. We investigated a life-threatening primary lung carcinoma with distant metastasis in a 73-year-old transgender woman, which posed unique challenges due to the advanced stage of the disease at presentation. The patient exhibited nonspecific musculoskeletal and neurological symptoms resulting from the primary lung carcinoma metastasizing to her liver, bones, and brain. We evaluated various imaging modalities that aided in determining the disease's severity and identifying complications related to metastasis. Although these efforts can offer symptomatic relief, the overall prognosis remains poor when metastasis spreads to multiple organs, particularly the brain, as remission may no longer be attainable.

## Introduction

Lung cancer is the leading cause of cancer-related deaths in the United States, with nearly two million new cases in 2022 [1]. Tobacco cigarette smoking is a well-known risk factor for lung cancer development. Non-small cell lung cancer (NSCLC) accounts for approximately 87% of cases, with nearly 40% attributed to the adenocarcinoma subtype [2]. Most patients with advanced disease initially present with symptoms; 40% have metastatic disease affecting the brain, liver, adrenal glands, or bones [3]. Metastatic disease in distant organs often indicates late-stage disease with a poor prognosis. Patients typically experience complex, nonspecific symptoms that are challenging to distinguish, especially when brain metastases are present. Despite advances in medical technologies and therapeutic approaches that aim to improve diagnosis and extend survival time, brain metastatic cancer is associated with higher complication rates and can even become a source of new malignancies.

This case report discusses the prolonged symptom burden and suboptimal symptom control associated with advanced metastatic cancer. In light of these challenges, shared medical decision-making becomes increasingly important, particularly in advanced care planning, as it necessitates active participation from patients and their families. Integrating palliative care and addressing support systems are essential to enhancing patients' quality of life.

## Case presentation

A 73-year-old transgender woman, recently diagnosed with metastatic lung adenocarcinoma, was admitted to our hospital from a skilled nursing facility (SNF) due to acute hypoxic respiratory failure and sepsis. One week prior, she had been hospitalized at a nearby medical facility for altered mental status, peripheral neuropathy, constipation, and lower back pain.

The patient had a medical history significant for hypertension, hyperlipidemia, coronary artery disease with two stents previously placed, and chronic low back pain following laminectomy. Her medications included oxycodone as needed, daily atorvastatin, metoprolol, enalapril, aspirin, and estrogen. She had been recently discharged with levetiracetam, gabapentin, and dexamethasone. She smoked one pack of cigarettes per day for 40 years.

**Figure 1:**
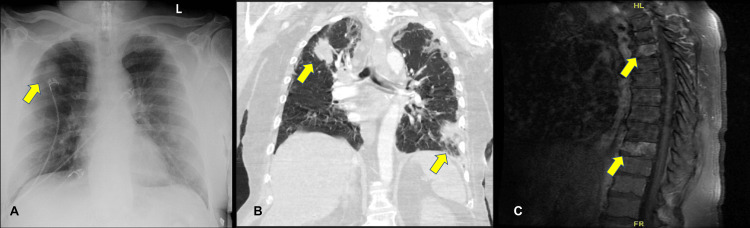
Results of imaging studies done on the patient A. Chest X-ray with a visible mass on the right upper lobe. B. CT scan of the chest. C. MRI of the thoracic spine. CT, computed tomography; MRI, magnetic resonance imaging

A chest radiograph revealed a right upper pulmonary mass (Figure 1A), prompting a chest computed tomography (CT) scan (Figure 1B), which identified irregular lesions in the right upper and left lower lobes, suggestive of neoplasm. Further examination disclosed osseous metastases in multiple vertebral bodies, including the left iliac bone, on magnetic resonance imaging (MRI) of the thoracic and lumbar spines (Figure 1C).

**Figure 2:**
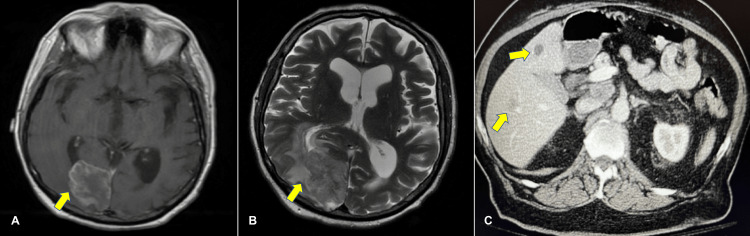
Brain MRI images A. MRI of the brain, axial view. B. MRI of the brain, horizontal view. C. CT scan of the abdomen with two lesions in the liver. CT, computed tomography; MRI, magnetic resonance imaging

A CT-guided core biopsy of the right lung confirmed primary adenocarcinoma of the lung. To assess potential metastasis, an MRI of the brain was obtained (Figures 2A, 2B), which revealed a large lobulated irregularly shaped intraaxial mass in the right parietal-occipital lobe, accompanied by extensive vasogenic edema and mass effect within the left cerebellar hemisphere. Additionally, the examination identified left eye ptosis. The care team promptly initiated levetiracetam and dexamethasone therapy to prevent vasogenic edema and seizures. Abdomen CT scans (Figure 2C) also detected liver lesions consistent with metastasis.

A neurosurgeon was consulted. Stereotactic-guided decompression of right occipital craniotomy removed 90% of the metastatic tumor. The pathology results confirmed metastatic non-small cell carcinoma of the lung. Postoperative radiation therapy to the tumor bed was planned on an outpatient basis. However, the day after discharge to the SNF, she experienced respiratory distress with an oxygen saturation slightly above 80% on room air. She tested positive for coronavirus disease 2019 (COVID-19) and was subsequently admitted to our facility for acute hypoxic respiratory failure and sepsis.

Upon arrival at our emergency department, her physical examination revealed a temperature of 38.2°C, a heart rate of 129 beats/min in sinus tachycardia, blood pressure readings of 108/63 and 92/53 mmHg, and a respiration rate of 36 breaths/min with labored breathing. Her oxygen saturation level at 4 L of oxygen was 95%. She was awake, alert, oriented, fatigued, and in moderate distress. Her head appeared normocephalic, with staples present in the posterior scalp without erythema or drainage. No visual field defects or ptosis were observed. Auscultation revealed bibasilar rales without tenderness, and no heart murmurs were detected. The patient exhibited active bowel sounds. Pedal edema was absent upon inspection, motor strength was normal in the upper extremities but weak in the lower extremities, and a loss of hand-eye coordination was observed, with sensations intact. A gait assessment was not performed due to the patient's weakness and reported imbalance.

The initial analysis in the emergency department (Table 1) showed 15,600 leukocytes with increased neutrophils, elevated levels of lactic acid, C-reactive protein, aspartate transaminase, troponin, D-dimer, and serum urea nitrogen, and low sodium and chloride levels. The COVID-19 test result was positive. Her arterial blood gas indicated respiratory alkalosis. Additionally, her chest X-ray revealed scattered small opacities in the lungs, suggestive of atypical pneumonia and a mass in the right upper lobe. Her electrocardiogram demonstrated sinus tachycardia without acute ischemic changes.

**Table 1:** Patient laboratory workup results

Parameter	Values	Reference Values
White blood cells (x10^3^/µL)	15600 µL	4000-10500 µL
Neutrophils	94.3%	34.0-71.0%
Lactic acid	3.0 mmol/L	0.4-2.0 mmol/L
C-reactive protein	36 mg/dL	<0.3 mg/dL
Aspartate transaminase	39 units/L	15-37 units/L
Troponins	61 ng/L	<54 ng/L
D-dimer	4855 ng/mL	0-529 ng/mL
Sodium	129 mmol/L	136-145 mmol/L
Chloride	93 mmol/L	98-107 mmol/L
Serum urea nitrogen	24 mg/dL	7-18 mg/dL

We initiated the sepsis protocol, administering intravenous fluid resuscitation, broad-spectrum antibiotics, and azithromycin for atypical pneumonia. The patient was admitted to the progressive care unit for continuous monitoring and evaluation. We continued her home medications, including levetiracetam and dexamethasone, and treated her with remdesivir and inhalation therapies.

**Figure 3:**
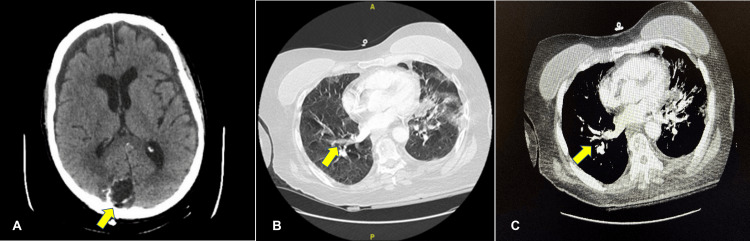
Post-treatment CT images of the patient's brain and chest A. CT scan of the brain post right occipital craniotomy. B. CT angiography of the chest with subsegmental pulmonary emboli in the right upper lobe. C. CT angiography of the chest axial view of the pulmonary emboli. CT, computed tomography

During her stay in the unit, the patient experienced episodes of confusion and disorientation. A repeat brain CT scan (Figure 3A) revealed irregular linear hyperdensities surrounding the surgical cavity, which could represent an acute bleed with diffuse vasogenic edema over the parietal convexity. Chest CT angiography (Figures 3B, 3C) was performed due to hypoxemia and tachycardia, revealing a small-volume pulmonary embolus (PE) without right heart strain. A venous ultrasound of the lower extremities was negative for deep vein thrombosis (DVT). The medical team faced a challenging decision regarding whether to initiate anticoagulation for PE, considering the recent craniotomy and the high suspicion of acute bleed seen in the brain CT. After consulting the neurosurgeon, we decided to defer anticoagulation due to the high risk of intracranial hemorrhage.

Additional laboratory tests identified positive blood *Pseudomonas* results, which narrowed the antibiotic requirement. The patient developed atrial fibrillation (Afib) with a rapid ventricular response (RVR) controlled with diltiazem. Her condition remained stable during the hospital stay. We discussed the option of an inferior vena cava (IVC) filter with the patient, but she declined further procedures. She expressed her desire to go home and be with her family multiple times, acknowledging her current medical condition and stating her preference to stay at home peacefully with her family. We consulted palliative care, and the family participated in discussions about the patient's goals of care. We made a shared decision, and the patient was discharged to hospice care.

## Discussion

Lung cancer remains the most common cancer worldwide [4], with smoking continuing as the leading risk factor for its development and the primary cause of death in both men and women [5]. Lung cancer is classified as either small-cell lung cancer (SCLC) or NSCLC. In this case, the patient, a known cigarette smoker, had NSCLC, the most common lung neoplasm [6], accounting for 87% of cases. Nearly 40% of patients are diagnosed with metastatic disease at presentation [3]. NSCLC is further classified as adenocarcinoma, large cell carcinoma, and squamous cell carcinoma based on histopathological examination [6]. The most common sites of distant metastases include the brain, liver, adrenal glands, and bones [3]. Brain metastasis accounts for half of the solid tumors [7], and approximately 20% to 30% of patients present with bone metastasis from a primary lung neoplasm [8].

Long-term use of hormone therapy in a transgendered woman may increase the risk of breast, testicular, and prostate cancers [9,10] with an increase in mortality due to malignancies of the lung [11]. Although sex-hormone receptors are mainly found in reproductive organs, they can also be found in other non-reproductive organs (e.g., the lung and the brain) which can increase the risk for tumor development [12]. Also, the cancer care of transgendered patients can be technically challenging due to stigma and discrimination [13] resulting in late stages of the disease and decreased survival.

The patient's brain MRI, chest CT angiography, and abdominal CT scan revealed that the primary lung cancer had metastasized to the brain, liver, and several vertebral bodies. Distant metastases typically result from vascular and lymphatic invasion. Patients with distant metastasis generally face a poor prognosis and high mortality [14] due to the significant associated complications, including vasogenic edema, seizures, venous thromboembolism [15], fractures, pain [16], pleural effusion, shortness of breath, and severe infections [3].

The lung cancer diagnosis was initially made with a chest radiograph showing a right upper lung mass. A chest CT scan, commonly used to describe the tumor extent better, was subsequently performed. The diagnostic accuracy of a CT scan (56% to 89%) is similar to that of an MRI (53%-93%) [17]. However, MRI is considered a superior modality for detecting brain metastases due to its enhanced contrast resolution. Distinguishing between primary and secondary neoplasms is essential. Tissue sampling is often necessary to confirm the diagnosis because masses seen on imaging may represent a nonneoplastic lesion. The core biopsy performed in this case was consistent with lung adenocarcinoma, predominantly seen in smokers and the most common histologic subtype [18].

Metastatic brain lesions can vary in symptom presentation depending on the affected areas. Although our patient experienced episodes of confusion and disorientation, she remained awake, alert, and oriented for the most part. She had ptosis at the initial presentation at the other facility, which was not observed under our care. She demonstrated impaired hand-eye coordination bilaterally. These symptoms were likely secondary to the effect of edema in the involved area. Headaches, nausea, vomiting, and imbalances were also observed. Angiogenic factors secreted by the tumor cause fluid extravasation into the brain parenchyma [15], leading to the clinical findings observed in our patient.

Treatment modalities depend on the stage and extent of the disease. Dexamethasone was administered in our case to minimize fluid retention and control symptoms. Corticosteroids are the mainstay therapy for vasogenic edema, with improvements in neurological functioning usually observed after 24 to 72 hours [15]. The mass effect of brain tumors can cause seizures. Although our patient did not present with seizures, levetiracetam was initiated to prevent any epileptogenic symptoms. Adjuvant radiotherapy was also planned postoperatively. Patients who underwent surgical resection combined with whole-brain radiotherapy experienced better overall survival and quality of life [7].

Multiple risk factors, including age, immobility, malignancy, corticosteroid use, and postoperative states, contributed to our patient's susceptibility to COVID-19 pneumonia, bacteremia, and PE, leading to acute hypoxic respiratory failure. The high levels of procoagulant proteins released by cancer cells cause the formation of thrombin and fibrin, which can result in venous thromboembolism (VTE) [19]. Patients with tumor cells have a 20% to 30% incidence of developing VTE [15,19]. Similarly, the patient's history of estrogen use could potentially be associated with VTE development.

The medical team faced a challenging decision of whether to start VTE treatment for the recently discovered low-volume PE versus increasing the risk of developing an intracranial bleed. The highly suspicious acute bleed reported in the repeated CT scan of the brain and the patient's recent craniotomy performed one week prior led to a joint decision to withhold anticoagulation because they believed that the risk of life-threatening intracranial hemorrhage was high in this patient. Anticoagulation is generally contraindicated in patients with recent neurosurgery within two weeks after the operation [20]. The patient had a craniotomy one week before presenting at our hospital, and although IVC filter placement was discussed with the patient, she ultimately refused further procedures. Intermittent pneumatic calf compression was continued during the hospital stay. It should be noted that IVC filter placement is still associated with an increased risk of DVT [19].

Later, the patient developed a new-onset Afib with RVR, treated with diltiazem. The patient's cancer-related comorbidities, including sepsis, hypoxia, recent surgery, electrolyte abnormalities, and metabolic disorders, could have triggered the occurrence of new-onset Afib.

Our patient and her family suffered psychological and emotional distress after the advanced cancer diagnosis. In anticipation of the negative impact of the diagnosis, the medical team integrated a patient and family-centered approach to promote well-being and improve their quality of life. The care team considered the patient’s preferences and treatment decisions. They also engaged in enhanced provider face-to-face communication among family members. A patient’s medical needs are foremost alongside their psychosocial needs.

Despite advances in cancer treatment, lung cancer has a poor prognosis, especially when metastasizing to the brain. This situation significantly burdens the patient and caregivers, resulting in distress and poor quality of life. Palliative and hospice care were integrated into the patient's care plan, fulfilling an exceptional and challenging role in providing support and resources needed for the patient and family to navigate such difficult times. They helped address the complex medical decisions required to preserve the quality of life without inappropriate prolongation, ultimately fulfilling the patient's wish to be with her family and loved ones. It is essential to note that the patient's preference for decision-making was met and that no further testing or procedures were performed.

## Conclusions

We discussed a challenging case of a transgender woman who initially presented with musculoskeletal and nonspecific neurological concerns and multiple complex medical conditions. A lung tumor identified from a simple chest radiograph revealed a life-changing event for the patient and her family. The metastatic non-small cell carcinoma of the lung is almost invariably associated with a poor prognosis and limited long-term survival. Various imaging modalities were utilized to assess the severity of the disease and identify complications associated with metastasis. Immediate surgical and pharmacological interventions were employed to alleviate symptoms. However, several complications, such as infections, pulmonary thrombosis, hypoxic respiratory failure, and Afib, arose from her complex medical conditions, ultimately requiring a shared medical decision to preserve the patient's and family's quality of life.
